# Polyamine Function in Plants: Metabolism, Regulation on Development, and Roles in Abiotic Stress Responses

**DOI:** 10.3389/fpls.2018.01945

**Published:** 2019-01-10

**Authors:** Dandan Chen, Qingsong Shao, Lianghong Yin, Adnan Younis, Bingsong Zheng

**Affiliations:** ^1^State Key Laboratory of Subtropical Silviculture, Zhejiang A&F University, Hangzhou, China; ^2^Department of Traditional Chinese Medicine, Zhejiang A&F University, Hangzhou, China; ^3^Institute of Horticultural Sciences, University of Agriculture, Faisalabad, Pakistan

**Keywords:** polyamines, flowering, embryonic development, senescence, abiotic stress

## Abstract

Polyamines (PAs) are low molecular weight aliphatic nitrogenous bases containing two or more amino groups. They are produced by organisms during metabolism and are present in almost all cells. Because they play important roles in diverse plant growth and developmental processes and in environmental stress responses, they are considered as a new kind of plant biostimulant. With the development of molecular biotechnology techniques, there is increasing evidence that PAs, whether applied exogenously or produced endogenously via genetic engineering, can positively affect plant growth, productivity, and stress tolerance. However, it is still not fully understood how PAs regulate plant growth and stress responses. In this review, we attempt to cover these information gaps and provide a comprehensive and critical assessment of the published literature on the relationships between PAs and plant flowering, embryo development, senescence, and responses to several (mainly abiotic) stresses. The aim of this review is to summarize how PAs improve plants' productivity, and to provide a basis for future research on the mechanism of action of PAs in plant growth and development. Future perspectives for PA research are also suggested.

## Introduction

Polyamines (PAs) are low molecular weight aliphatic nitrogenous bases containing two or more amino groups, and they have potent biological activity (Xu et al., 2009; Vuosku et al., 2018). They are widely distributed in eukaryotic and prokaryotic cells (Liu et al., 2017; Mustafavi et al., 2018). In living organisms, PAs mainly exist in free (F-PAs), covalently conjugated (CC-PAs) or non-covalently conjugated (NCC-PAs) forms (Gholami et al., 2013). The CC-PAs can be divided into perchloric acid-soluble covalently conjugated polyamines (PSCC-PAs) and perchloric acid-insoluble covalently conjugated polyamines (PISCC-PAs).

In higher plants, PAs are mainly present in their free form. Putrescine (Put), spermidine (Spd), and spermine (Spm) are the main PAs in plants, and they are involved in the regulation of diverse physiological processes (Xu et al., 2014b; Mustafavi et al., 2018), such as flower development, embryogenesis, organogenesis (Xu, 2015), senescence, and fruit maturation and development. They are also involved in responses to biotic and abiotic stresses (Vuosku et al., 2012; de Oliveira et al., 2016; Reis et al., 2016; Mustafavi et al., 2018).

Free polyamines covalently combine with a small molecular substance, such as a phenolic compound and a derivative thereof in the amide bond to form a binding PA, which is also known as a PSCC-PA. The phenolic compound may be hydroxy cinnamic acid, coumaric acid, caffeic acid, or ferulic acid (Luo et al., 2009; Martin-Tanguy, 2010). This kind of PA forms the largest pools of PAs in plants (Kusano et al., 2008; Bassard et al., 2010). Many studies have confirmed that PSCC-PAs act as secondary metabolites, and participate not only in the local allergic reaction of plants against external infestation (Kumar et al., 1997), but also in plant morphogenesis (de Oliveira et al., 2016; De Oliveira et al., 2018; Mustafavi et al., 2018).

Free polyamines covalently bind to biomacromolecules, such as proteins, nucleic acids, uronic acids, or lignin by ionic and hydrogen bonds to form bound PAs, also known as PISCC-PAs. In the physiological pH range, F-PAs are fully protonated and positively charged, and can electrostatically combine with negatively charged biomacromolecules, such as acidic proteins, membrane phospholipids, and nucleic acids in the organism to become NCC-PAs (Igarashi and Kashiwagi, 2015). The NCC-PAs are associated with the regulation of enzyme activity, DNA replication, gene transcription, cell division and membrane stability, and have a wide range of biological functions in plant growth and development. Generally, the more the amino groups, the stronger the physiological activity.

Recent studies using exogenous PAs, PA synthesis inhibitors, and transgenic methods have intensively investigated the role of PAs in plant development and their mechanism of action. Such studies have shown that PAs are closely associated with plant growth, the stability of nucleic acids and membrane structure, stress resistance, and even plant survival (Agudelo-Romero et al., 2013; Pál et al., 2015; Sequeramutiozabal et al., 2016).

In this review, we provide a comprehensive and critical assessment of the published literature on the relationship between plant PAs and plant growth and development. We summarize recent research on the effects of PAs on the development of plants from flowering to embryonic development to senescence, and explore their roles in the responses to several stresses. The aim of this paper is to reveal the roles that PAs play in plant growth and development and provide a basis for future research on the mechanism of action of PAs in plant growth and development. We also discuss the ways in which exogenous PAs can be used regulate and promote plant growth and development in production.

## Distribution and Metabolism of PAs in Plants

### PAs Distribution

Polyamines are ubiquitous in eukaryotic and prokaryotic cells (Liu et al., 2016, 2017), and are found even in plant RNA viruses and plant tumors. They have potent biological activities. There are numerous forms of PAs. In higher plants, PAs are predominantly present in their free form. The most common PAs in higher plants are Put, Spd, Spm, thermospermine (Tspm) (Kim et al., 2014; Sobieszczuk-Nowicka, 2017; Takahashi et al., 2017b), and cadaverine (Cad) (Regla-Márquez et al., 2015; Nahar et al., 2016) (Table 1). Other PAs are found only in certain plants or under special conditions.

**Table 1:** Polyamine structure and distribution.

**Name**	**Structure**	**Molecular formula**	**Source**
Agm	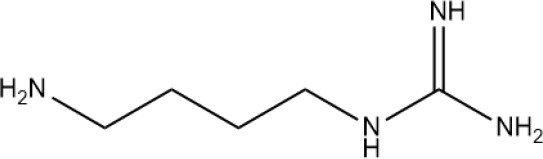	C_5_ H_14_N_4_	ubiquitous
Put	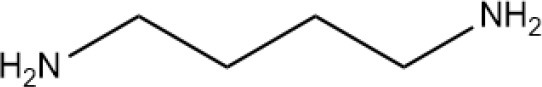	C_4_ H_12_N_2_	Ubiquitous
Spd	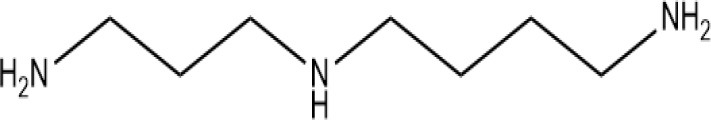	C_7_H_19_N_3_	Ubiquitous
Spm	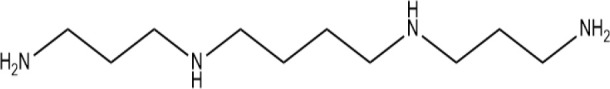	C_10_H_26_N_4_	Ubiquitous
Cad	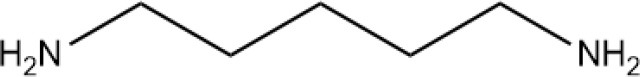	C_5_H_14_N_2_	Legume plants
Tspm	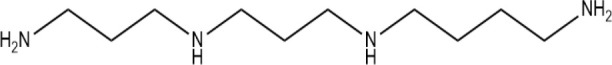	C_10_H_26_N_4_	–

Polyamines show tissue- and organ-specific distribution patterns in plants. For example, the most abundant PA in leaves was found to be Put, and its levels were three times higher than those of Spd and Spm, whereas Spd was found to be the most abundant PA in other organs (Takahashi et al., 2017b). Different types of PAs also show different localization patterns within cells. In carrot cells, Put was found to accumulate in the cytoplasm, and Spm in the cell wall (Cai et al., 2006). The distribution patterns of PAs may be related to their unique functions. In general, more vigorous plant growth and metabolism is associated with greater PA biosynthesis and higher PA contents (Zhao et al., 2004; Cai et al., 2006).

### Polyamine Biosynthesis

**Figure 1:**
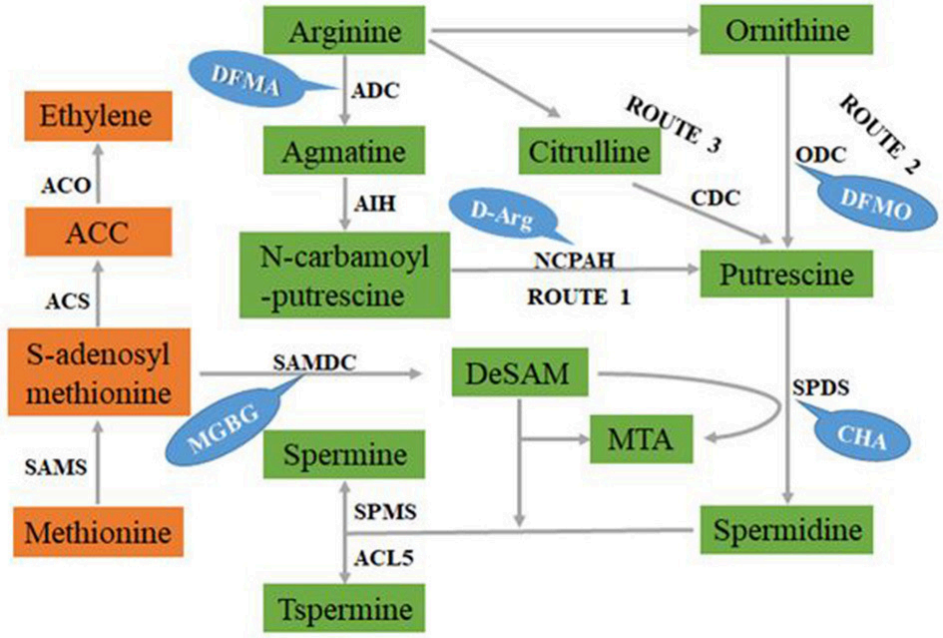
The pathway of PAs biosynthesis in plant. The orange part is the ethylene synthesis pathway, and the green part is the polyamine synthesis pathway (There are three routes of putrescine synthesis, route 1, route 2, and route 3), and the blue part is the corresponding enzyme inhibitor.

Putrescine is the central product of the common PA biosynthetic pathway. It contains two amino groups and is a synthetic precursor of Spd and Spm (Xu et al., 2009). There are three different routes of Put biosynthesis in plants (Figure 1). In the first route, the No. 8 carbon atom is removed from arginine (Arg) by arginine decarboxylase (ADC) to form agmatine (Agm) and CO_2_; the No. 2 nitrogen atom is removed from Agm to form N-carbamoyl Put (NCPA) and NH_3_; and then NCPA is hydrolyzed by N-carbamoylputreseine amidohydrolase (NCPAH) and its carbamoyl group is removed to form Put, CO_2_, and NH_3_. This is the main Put synthesis pathway in plants (Docimo et al., 2012; Pegg, 2016). In the second route, ornithine (Orn) is produced from Arg by arginase; and then ornithine decarboxylase (ODC) removes the carboxyl group of the no.1 carbon atom of Orn to form Put and CO_2_ (Docimo et al., 2012; Pegg, 2016). The *ODC* gene has been lost from *Arabidopsis thaliana* and many members of the *Brassicaceae* (Hanfrey et al., 2010), indicating that the ornithine pathway is not essential for normal growth. In the third route, Arg is first converted into citrulline (Cit), which is then decarboxylated by citrulline decarboxylase (CDC) to form Put (Han, 2016; Ouyang et al., 2017; De Oliveira et al., 2018). To date, the Cit pathway has only been found in sesame, and so the first two pathways are more common in plants. The activities of ADC and ODC can be inhibited by the irreversible competitive inhibitors difluoromethylarginine (DFMA) and difluoromethylornithine (DFMO), respectively (Grossi et al., 2016; Yamamoto et al., 2016). Spermidine and Spm are produced from Put and aminopropyl residues, which are gradually provided by methionine (Vuosku et al., 2018) (Figure 1).

### Polyamine Catabolism

**Figure 2:**
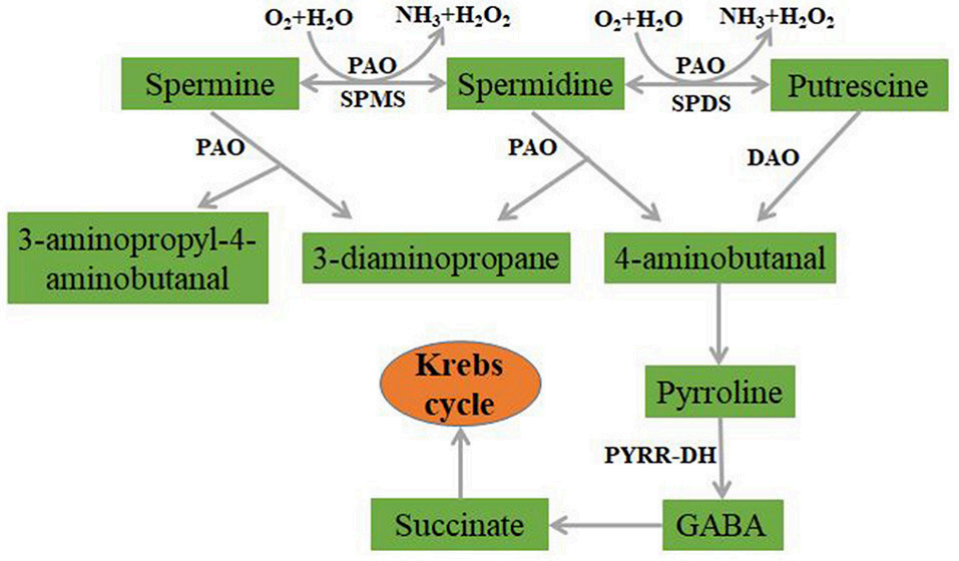
The pathway of polyamine catabolism in plant.

The catabolism of PAs in plants is mainly dependent on the action of amine oxidases. The known amine oxidases include diamine oxidase (DAO) and PA oxidase (PAO) (Figure 2). Diamine oxidase, which relies on Cu^2+^ and pyridoxal phosphate as its cofactors, catalyzes the formation of H_2_O_2_, ammonia, and 4-aminobutanal from Put. Then, 4-aminobutanal undergoes cyclization to form pyrroline (PYRR), which is converted into γ-aminobutyric acid (GABA) by the action of pyrroline dehydrogenase (PYRR-DH). Then, GABA is further converted into succinate, which enters the Krebs cycle. Dicots contain high contents of DAO, but its encoding gene has been found in only a few species (Cona et al., 2006). Unlike DAO, PAO is linked to flavin adenine dinucleotide (FAD) by non-covalent bonds and is found at high levels in monocots (Takahashi et al., 2017a; Hao et al., 2018). Its substrates are advanced PAs, such as Spd, Spm, and Tspm. There are multiple PAO families in many plants (Liu et al., 2014; Takahashi et al., 2017a). Some PAOs catalyze the production of metabolic end-products of PAs; for example, the wheat PAO oxidizes Spd and Spm to form 4-aminobutanal,3-aminopropyl-4-aminobutanal,1,3-diaminopropane (Dap) and H_2_O_2_ (Cona et al., 2006; Liu et al., 2014). Some PAOs catalyze the reverse reaction of PA synthesis in the PA back-conversion pathway (PBCP) (Liu et al., 2014; Takahashi et al., 2017a). Del Duca and Tassoni et al. found that exogenous Spd applied to *Helianthus tuberosis* and *A. thaliana* was transformed into Put (Tassoni et al., 2000). In *Arabidopsis*, PAO1 and PAO4 were able to convert Spm to Spd; and PAO2 and PAO3 catalyzed the production of Spd from Spm and then produced Put (Moschou et al., 2008). The PAO2 of *Brachypodium distachyon* catalyzed the conversion of Spm or Tspm to Spd, and Spd to Put, with Spd as the preferred substrate. In contrast, BdPAO3 preferentially utilized Spm as the substrate and catalyzed the conversion of tetraamines to Spd (Takahashi et al., 2017a) (Figure 2).

The metabolism of PAs in plants is closely connected to many other metabolic pathways. The H_2_O_2_ produced by PA oxidation functions in the signal transduction process of plants during biotic and abiotic stress responses (Freitas et al., 2017; Mellidou et al., 2017), and affects stomatal closure induced by abscisic acid (ABA) (Cona et al., 2006; Tun et al., 2006; An et al., 2008). S-adenosylmethionine (SAM) in the PA biosynthetic pathway is also a precursor for ethylene synthesis (Figure 1), and studies have demonstrated that PAs synthesis competes with ethylene synthesis (Lasanajak et al., 2014). In addition, the metabolism of PAs is related to the production of NO (Pál et al., 2015), which is an essential signaling component for plant growth (Agurla et al., 2017). Therefore, the roles of PAs in plant growth and development and the mechanisms underlying their function can be explored by studying the relationship between PA metabolism and plant hormones, and the effects of PA metabolism on plant signaling substances.

## Polyamines and Plant Development

### Polyamines and Flowering

After a period of vegetative growth, higher plants enter a period of reproductive growth; that is, leaf bud tissue changes its physiological state to become flower bud tissue, and then develops into a floral organ. This process is called flower bud differentiation (Guo et al., 2015). Flower bud differentiation is a complex process of morphogenesis. It is triggered by various factors, such as photoperiod, vernalization, nutrition, and water status, and is accomplished by the interaction and coordination of hormones and PAs (Xu, 2015).

Polyamines are considered to be a class of growth regulators in plants (Xu et al., 2014b). Many studies have shown that exogenous PAs and PA synthesis inhibitors can affect flower bud differentiation. Exogenous PAs were shown to accelerate the process of flower bud differentiation, and high PA contents in apical buds were beneficial for the initiation and maintenance of flower bud differentiation in *Chrysanthemum* (Xu, 2015) (Table 3). In *Arabidopsis*, PAs were found to be more abundant in flowers than in any other organ, and the addition of exogenous PAs to poorly flowering plants significantly promoted their flowering response (Applewhite et al., 2010) (Table 3). The application of Spm (10 ppm) improved flower quality and extended vase life by 3 days in cut rose flowers (Tatte et al., 2015). Lower contents of PAs (mainly Put and Spd) in rapeseed were found to be conducive to the initiation of flower bud differentiation, and an increased PAs content was beneficial for flower bud development. Earlier peaking of PA contents in tissues led to earlier bolting time (Ai et al., 2011). Similar results were observed in *Dendrobium nobile*, where plants with higher levels of Put and Spd in the leaves had more flower buds, more flowers, and a larger mean floral diameter (Li et al., 2014). The recombinant proteins of GtSPDS and GtSPMS from *Gentiana triflora* (homologs of two *Arabidopsis* PA biosynthetic enzymes) had SPDS and SPMS activity, respectively. The expression levels of *GtSPDS* and *GTSPMS* transiently increased from the vegetative to the reproductive growth phase, and overexpression of these genes hastened flowering (Applewhite et al., 2010; Imamura et al., 2015) (Table 2).

**Table 2:** Genes related to polyamines on regulating plant growth.

**Plant species**	**Gene**	**Effect**	**Outcome**	**Citation**
*Citrus sinensis*	*CsPAO3*	Overexpression of *CsPAO3* in tobacco, Spd and Spm↓, Put↑	*CsPAO3* plays a potential role in PAs back conversion	Wang and Liu, 2015
*Gossypium hirsutum* L	*GhPAO3*	In transgenic *Arabidopsis*(*GhPAO3*), Spm↓, Put↑	*GhPAO3* plays a potential role in the conversion of Spd and Spm	Cheng et al., 2017
Transgenic rice	*OsSAMDC2*	Transcript levels of *OsSAMDC1, OsSAMDC2*, and *OsSAMDC4* were all reduced in transgenic rice, Spd, Spm, and PAs oxidase activity↓	Spd and Spm are essential for maintenance of normal plant growth, pollen viability, seed setting rate, grain yield and stress tolerance in rice	Chen et al., 2014
Transgenic tomato	Mouse *ODC*	Put, Spd and Spm↑, ethylene, respiration rate and physiological loss of water↓	Enhances fruit quality in tomato	Pandey et al., 2015
*Gentiana triflora*	*GtSPDS* or *GtSPMS*	The expression levels of *GtSPDS* and *GtSPMS* increased transiently during vegetative to reproductive growth phase	Hasten flowering	Imamura et al., 2015
*Pyrus betulaefolia*	*PbrMYB21*	Modulate the PAs synthesis by regulating the *ADC* expression	Plays a positive role in drought tolerance	Li et al., 2017
*Medicago falcata*	*MfERF1*	Up-regulates the genes associated with PAs synthesis and catabolism, promotes PAs turnover, antioxidant protection	Confers cold tolerance	Zhuo et al., 2018

*↓: Indicates a decrease in substance content or enzyme activity; ↑: Indicates an increase in substance content or enzyme activity*.

**Table 3:** Effects of polyamines on plant growth and development.

**Plant species**	**Polyamine treatment**	**Effect**	**Outcome**	**Citation**
*Arabidopsis thaliana*	Spd (0.3 or 3 mM) CHA + DFMO (4 mM)	Inhibitors preventing bolting and flowering, exogenous PAs to poorly flowering plants can significantly add to their flowering response	PAs promote flowering	Applewhite et al., 2010
*Dendranthema morifolium*	Spd (0.1 mM/L)	Significantly affect endogenous polyamines (Spd, Spm) and endogenous hormones (IAA, ZR, IPA, GA)	Accelerate the process of flower bud differentiation	Xu, 2015
Wheat	Spd or Spm (1 mM)	In wheat grains, endogenous Spd, Spm, ABA, and IAA contents ↑, ETH content↓	Increased the grain filling rate and the grain weight	Liu et al., 2013
Sugarcane	Put (500 μM)	Somatic embryos in embryogenic callus↑	Induces somatic embryo development	Reis et al., 2016
Seedless grapevine	PAs (0.3–3 mM)	Embryo germination rate↑	Efficiency of embryo rescue *in vitro*↑	Jiao et al., 2017
Indica rice	Put (30 mg/l)	Spm and Spd contents↑, affect the expression levels of *ADC1* gene and *SAMDC* gene	Improve the growing state and the callus embryogenic traits	Tan et al., 2017

*↓: Indicates a decrease in substance content or enzyme activity; ↑: Indicates an increase in substance content or enzyme activity*.

Applying polyamine synthase inhibitors to the growth medium reduced the Spd content in Arabidopsis, and almost completely inhibited bolting and flowering. When the plants were transferred to medium without inhibitors, bolting and flowering were restored (Applewhite et al., 2010; Xu et al., 2014b; Xu, 2015) (Table 3). However, feeding Spd via the roots under permissive flowering conditions resulted in delayed flowering in *Arabidopsis* (Applewhite et al., 2010; Ahmed et al., 2017). Overexpression of *ADC* resulted in Put accumulation in the leaves, and plants showed a dwarf and delayed-flowering phenotype (Ahmed et al., 2017). Endogenous Put was found to be closely related to IAA and gibberellin (GA) contents, and high levels of Put and Spd were not conducive to the accumulation of IAA and GA (Xu, 2015). The effects of exogenous PAs and PA synthesis inhibitors on GA were mainly observed at the inflorescence differentiation and floret differentiation stages (Xu, 2015). Both the dwarf and delayed-flowering phenotypes were alleviated by spraying leaves with GA. Under short-day conditions, exogenous Spd significantly promoted PAO activity and lignin synthesis during flower bud differentiation. D-arginine inhibits flower bud differentiation, and reduces PAO activity and lignin synthesis (Xu et al., 2014a). Lignin is a secondary metabolite in plant growth and development, and it is of great significance in the growth, differentiation, and resistance of plant cells (Smita and Upendranath, 2008).

### Polyamines and Embryo Development

Polyamines have typical polycation characteristics. They bind to negatively charged nucleic acids, proteins, and phospholipids by ionic and hydrogen bonds through their amino and imino groups, and participate in zygote polarity establishment, apical axis formation, cell layer differentiation, and establishment of the meristem (Cangahuala-Inocente et al., 2014; Tiburcio et al., 2014). Polyamines are generally regarded as regulators in the process of embryogenesis in both angiosperms and gymnosperms (de Oliveira et al., 2016), and an increase in PAs content is required for embryogenesis. Studies have shown that the normal development of plant embryos requires a well-maintained dynamic balance of PAs *in vivo*. The types and abundance of PAs vary among different stages of embryonic development, from the multi-cell proembryo, globular, heart-shaped, and torpedo stages to the cotyledon stage (Krasuska et al., 2013). It is possible to regulate nucleic acid synthesis and protein translation in both directions by applying exogenous PAs and PA synthesis inhibitors. This can affect the development of organelles, such as endoplasmic reticulum, plastids, and mitochondria, and the structures of microtubules (Vondráková et al., 2015).

Generally, efficient somatic embryogenesis and the growth of embryos into complete plantlets are closely related to the levels of endogenous hormones, such as IAA, cytokinins (Cyt), ethylene, ABA, and PAs. Many studies have shown that PAs play a vital role in inducing cell division and promoting regeneration in plant tissues and cell cultures (Minocha and Minocha, 1995; Yadav and Rajam, 1997; Vondráková et al., 2015). In general, PAs are more abundant in embryogenic callus and somatic and zygotic immature embryos than in mature and germinating embryos. Putrescine stimulates somatic embryogenesis, and reduced concentrations of Put and Spd result in fewer somatic embryos. In cultured *Panax ginseng* somatic embryos, the addition of PAs at different concentrations (10–1,000 μm) to induction or regeneration media affected the formation of embryogenic structures. A 5- and 4-fold increase in the number of embryogenic structures was obtained by adding Spd (1,000 μm) to induction and regeneration medium, respectively (Kevers et al., 2000). In the embryogenic suspensor mass (ESM) of *Norway spruce*, the Put and Spd contents were approximately equal at the early stage of proliferation, but after 4 weeks, the Spd level was significantly higher than the Put level (Vondráková et al., 2015). In a range of hybrid combinations of seedless grapevine, the addition of 3 mM Put, 0.5 mM Spd, or 0.3 mM Spm to the culture medium significantly promoted plantlet development or the embryo germination rate. This indicated that addition of appropriate amounts of PAs to the culture medium could significantly increase the efficiency of *in vitro* embryo rescue for seedless grapevine (Jiao et al., 2017) (Table 3). A study on embryo development in litchi showed that the contents of Put, Spd, and Spm were higher in normal ovules than in abortive ovules during embryonic development (Chen and Lv, 2000).

Other studies have used PA synthesis inhibitors to explore the roles of PAs in plant embryogenesis. The addition of PA biosynthesis inhibitors (DFMO and DFMA) to induction and regeneration media at all tested concentrations (10–1,000 μm) significantly reduced the number of *P. ginseng* somatic embryos (Kevers et al., 2000). The concentrations of Spm and Spd were 11 times and 3 times higher, respectively, in embryogenic callus than in non-embryogenic callus of *Coffea canephora*, but the Put content did not differ significantly between embryogenic callus and non-embryogenic callus. Exogenous PAs resulted in a 58% explant response for embryogenesis, compared with a 42% response in the control. The PA biosynthesis inhibitors DFMO and DFMA caused an 83% decrease in the embryogenic response (Kumar et al., 2008). These results were consistent with those of other studies (Bais and Sudha Gravishankar, 2001).

With the development of molecular biology techniques, genes encoding key enzymes in PA biosynthesis have been successfully isolated and cloned from plants, such as rice, tobacco, and *Arabidopsis*, and the corresponding mutants have been obtained by T-DNA insertion mutation (Su et al., 2012; Miller-Fleming et al., 2015). Analyses of these genes and signal transduction regulators in wild-type and mutant *Arabidopsis* revealed that one mutant had a blocked PA signal transduction pathway, which in turn affected cell division and differentiation (Gallois et al., 2013; Molesini et al., 2015).

### Polyamines and Plant Senescence

The activities of PA metabolic enzymes and PAs contents change throughout the stages of plant growth. In whole plants, endogenous PAs and PA synthetase activity were found to be highest in the meristem and growing cells, and lowest in senescent tissues. As leaves senescence, the chlorophyll content gradually decreases, and the activities of ADC and ODC decrease, while the activities of PAO and hydrolases, such as ribonuclease and protease increase rapidly. All of these changes can be inhibited by the application of exogenous PAs (Duan, 2000; Cai, 2009) A reduction in PA levels seems to be a significant prelude to senescence signals, or it may be that a decrease in PAs content is the senescence signal (Duan et al., 2006).

Exogenous Spd and Spm treatments can increase the PAs content in cut flowers, and delay their senescence and improve quality (Yang and He, 2001; Cao, 2010). In *Anthurium andraeanum*, the application of GA_3_ + Spm by spraying delayed the senescence of cut flowers stored at 20°C, and improved the quality of the inflorescences (Simões et al., 2018). Delayed leaf senescence was found to be associated with a higher Spm level, reduced reactive oxygen species (ROS) production, and increased NO levels (Sobieszczuk-Nowicka, 2017). Polyamines appeared to delay senescence by inhibiting ethylene biosynthesis (Woo et al., 2013; Anwar et al., 2015).

Gerbera flowers sprayed with 0.1 mM Spd or treated with 10 mM Spd in vase water showed delayed senescence, while those sprayed with 1 mM Spd, 10 mM Spd, 0.1 mM Spm, 1 mM Spm, or mixed solution of 0.1 mM each of Put, Spd, Spm showed accelerated senescence, with brown spots and yellowing of the petal rims starting from day 2 of treatment (Bagni and Tassoni, 2006). Legocka and Serafni-Fracassini et al. found that chlorophyll rapidly degraded and Put accumulated during senescence, while the exogenous addition of Spd or Spm inhibited protein degradation and reduced chlorophyll losses (Serafini-Fracassini et al., 2010; Cai et al., 2015). In peony, a PA synthesis inhibitor (0.1 Mm) extended the lifespan and delayed the senescence of cut flowers, while PAs shortened the lifespan and accelerated flower senescence (Han, 2016).

## Polyamines and Abiotic Stress Responses

### Polyamines and Temperature Stress

There are two major categories of temperature stress; low and high temperature stress. Low temperature stress can be further divided into cold stress and freezing stress. To date, few studies have focused on the physiological functions of PAs in plants under high temperature stress. High temperature stress significantly affected PA synthesis in the leaves of Chinese kale; after 6 days of high temperature treatment, the total PAs and Put contents had increased, but the increases were not sustained over longer treatment times (Yang and Yang, 2002). Under high temperature stress, PAs can promote photosynthesis, and increase the antioxidant capacity and osmotic adjustment ability of plants (Tian, 2012). Antioxidant enzymes can scavenge ROS to prevent membrane lipid peroxidation and stabilize membrane structure (Ouyang et al., 2017). Shao et al. reported that the heat tolerance of alfalfa was related to higher Spd contents and lower Put and Spm contents (Shao et al., 2015). The PAs have many different functions in plants, and the main physiological mechanisms of high temperature tolerance differ among plant species. This explains why the various PAs show different patterns of change in different plant species under high temperature stress (Shao et al., 2015).

Polyamines can bind to the phospholipid site of the cell membrane to prevent cytolysis and improve cold resistance (Li and He, 2012) (Table 2). However, there are several different viewpoints on the relationship between Put and plant chilling stress (Wu and Yuan, 2008). When sweet pepper and zucchini fruits were stored at chilling temperature, the Put content increased exponentially, accompanied by chilling damage. Storage under CO_2_ modified atmosphere reduced the extent of cold damage and inhibited the accumulation of Put, suggesting that Put accumulated as a result of chilling stress (Serrano et al., 1997, 1998). In contrast, Roy et al. proposed that Put accumulation caused chilling damage, and increased Spm may be a defense response to cold damage. They found that the Put, Spm, and Spd contents gradually increased in loquat fruit stored at low temperatures. The application of exogenous Spm maintained high levels of endogenous Spm and Spd, inhibiting Put accumulation and reducing chilling damage (Zhen et al., 2000; Roy and Wu, 2001). Another opinion was that Put may accumulate as a defense response of plants to chilling damage, because Put accumulation was found to be positively correlated with the cold resistance of plants (Wang et al., 2003b).

Sun et al. studied the effect of Put and D-Arg at different concentrations (0.5, 1.0, 1.5, and 2.0 mmol/L) on the physiological and biochemical indexes of *Anthurium andraeanum* under chilling stress at 6°C in winter. They found that Put application resulted in increased antioxidant enzyme activities, root activity, nitrogen metabolism, chlorophyll content, and proline content, and a decrease in malondialdehyde content. Treatment with 1.0 mmol/L Put had the strongest effect, and chilling damage was reduced by treatment with D-Arg (Sun et al., 2018b). Similar results were obtained for stevia plants, where PA supplementation increased their tolerance to cold conditions (Peynevandi et al., 2018). When an *SPDS* cDNA from *Cucurbita ficifolia* was introduced into *Arabidopsis* (Kasukabe et al., 2004), the transgenic plants exhibited a significant increase in SPDS activity and Spd content in leaves together with enhanced tolerance to various stresses including chilling and freezing (Groppa and Benavides, 2008). Recent studies have suggested that abiotic stress tolerance is mainly affected by the role of PAs in signal transduction rather than their accumulation (Pál et al., 2015).

### PAs and Water Stress

Most studies on the relationship between PAs and water stress have focused on drought resistance (Ebeed et al., 2017), and few have focused on waterlogging resistance. Polyamines (Spm, Spd, and Put) can regulate the size of the potassium channel and the size of pores in the plasma membrane of guard cells, thereby strongly regulating pore opening and closing. In this way, PAs can control water loss in plants (Liu et al., 2000). Many studies have shown that foliar application of Put at an appropriate level can trigger physiological processes and induce the biosynthesis of osmotic adjustment substances, such as free amino acids, soluble sugars, and proline. This may compensate for the negative impacts of drought stress on plant biomass and increase the quality and quantity of certain bioactive substances (Sánchezrodríguez et al., 2016; Mohammadi et al., 2018). In alfalfa, a Put treatment was shown to improve seed germination and increase all growth indexes (hypocotyl length, root and shoot fresh and dry mass) under drought stress caused by different concentrations of polyethylene glycol (PEG 4000), both *in vitro* and in a pot experiment (Zeid and Shedeed, 2006) (Table 4).

**Table 4:** Effects of polyamines on plant abiotic stress.

**Plant species**	**Stress**	**Polyamine treatment**	**Effect**	**Outcome**	**Citation**
*Alfalfa*	PEG (4,000)	Put (0.01 mM)	Germination, polysaccharide, protein, photosynthetic pigment contents and all growth criteria↑	Reduces the sensitivity of alfalfa to drought stress	Zeid and Shedeed, 2006
Wheat	Drought stress	PAs	Spd and Spm relieve the inhibition caused by drought stress, and Put has the opposite effect	Grain filling and drought resistance↑	Yang et al., 2016
*Agrostis stolonifera*	Drought stress	Spm (1 mM) Spd (5 mM)	Turf quality, relative water content, photochemical efficiency and membrane health↑, GA1, GA4, and ABA↑	Enhance the drought stress tolerance and growth of plant	Krishnan and Merewitz, 2017
*Thymus vulgaris* L.	Water stress	Put (20 mg/L)	Leaf water content, dry matter and antioxidant enzyme activities↑, cell injury indices↓	The negative impacts of drought stress on plants ↓	Mohammadi et al., 2018
*Panax ginseng*	NaCl (150 mM)	Spd (0.01, 0.1, 1 mM)	Chlorophyll degradation↓, Spd, Spm and the activities of enzyme scavenging system↑	Enhance salt tolerance	Parvin et al., 2014
*Zoysia japonica* Steud	Salt stress (200 mM)	Spd (0.3 mM)	Polyamine biosynthetic enzyme levels↑, H_2_O_2_ and MDA levels↓	Improved tolerance to salinity stress	Li et al., 2016
*Bakraii citrus* seedlings	NaCl (75 mM)	PAs (0.5–1, 0.5 mM Spd best)	The negative effects of salinity stress↓, growth parameters↑	Improve plant salinity tolerance	Khoshbakht et al., 2017
*Cucumis sativus*	NaCl (75 mM)	Spd (0.1 mM)	PAs, H_2_O_2_, SOD, POD and CAT↑, antioxidant defense↑, oxidative damage↓	Improve salt tolerance in cucumber seedlings	Wu et al., 2018
*Cerasus humilis* seedlings	Oxidative stress	Spd or Spm (0.2 mM)	The activities of ADC, ODC, SAMDC and antioxidant systems↑, endogenous free Put, Spd and Spm↑	Prevent oxidative damage induced by drought	Yin et al., 2014
Muskmelon	Ca(NO_3_)_2_ (80 mM)	GABA (50 mM)	The activities of ADC, ODC, SAMDC, PAO and DAO↑, Spd and Spm ↑, Put↓	Improve muskmelon seedling tolerance to Ca(NO_3_)_2_ stress	Hu et al., 2015
Wheat and Sunflower	CdCl_2_ or CuCl_2_ (1 mM)	PAs (0.1 mM)	Pevent the deleterious effect caused by Cd and Cu during plant development	Improve tolerance to heavy metal	Benavides et al., 2018

*↓: Indicates a decrease in substance content or enzyme activity; ↑: Indicates an increase in substance content or enzyme activity*.

The Arabidopsis mutant acl5/Spms, which cannot produce Spm, is hypersensitive to high salt and drought. This phenotype was cured by a Spm pretreatment but not by pretreatments with Put and Spd, suggesting that the drought-hypersensitivity of the mutant is due to Spm deficiency (Yamaguchi et al., 2007). A high Spm content and a high ratio of (Spd + Spm)/Put were associated with the drought resistance of mycorrhizal masson pine (Xu et al., 2009). Among the three main endogenous PAs, Spm was most strongly related to drought resistance apple (Liu et al., 2010). Similar results were obtained for cherry tomato (Montesinos-Pereira et al., 2015). However, Yang et al. found that Spd and Spm relieved the inhibitory effects of drought stress and promoted grain filling and drought resistance in wheat, while Put had the opposite effect (Yang et al., 2016) (Table 4).

The above results indicate that the function of PAs can differ among different plants and even different parts of the same plant, whether under osmotic stress or water stress (Sen et al., 2018). Therefore, the response of plants to exogenous PAs under osmotic stress and water stress will depend on the plant species.

### PAs and Salt Stress

Salt and drought stress are the two major abiotic stresses in agriculture, and both of them lead to reduced water potential in plants. Salinity is a complex environmental constraint. A high salt concentration reduces membrane integrity, decreases the activity of various enzymes, and impairs the function of the photosynthetic apparatus. Plants adapt to such unfavorable environmental conditions by accumulating low molecular-weight osmolytes, such as proline and PAs. The application of different types and concentrations of exogenous PAs has been shown to alleviate the effects of NaCl stress on various plants, and reduce damage (Verma and Mishra, 2005; Li et al., 2008) (Table 4). Plants rich in PAs usually show strong salt tolerance.

It has been suggested that the level of Spm in plants is an important indicator of salt tolerance (Li and He, 2012). The free, acid-soluble bound, and total Spm contents in leaf tissues of sunflower plants increased under 50, 100, or 150 mM NaCl treatments (Mutlu and Bozcuk, 2005). Exogenous PAs, especially Spm and Spd, resulted in increased reactive oxygen metabolism and photosynthesis, which improved plant growth and reduced the inhibitory effects of salt stress (Meng et al., 2015; Baniasadi et al., 2018). Similar results were obtained in a study on soybean seedlings (Wang and Bo, 2014). Li et al. produced a cucumber line with up-regulated *SAMDC* expression and down-regulated *ADC* and *ODC* expression, resulting in increased accumulation of Spd and Spm and decreased accumulation of Put under salt stress. As a result, the inhibition of plant growth under salt stress was alleviated in the transgenic seedlings (Li et al., 2011; Takahashi et al., 2017b). Several metabolic pathways are affected by Spm and Spd (Paul and Roychoudhury, 2017). Sun et al. showed that PAs and ABA together alleviated salt stress in grape seedlings (Sun et al., 2018a).

Recent studies have explored the relationship between PAs and plant drought resistance by using genetic engineering techniques. Malabika et al. transformed the oat *ADC* gene into rice, and found that the ADC activity, biological yield, and Put contents were higher in the transgenic rice and its progeny than in non-transgenic rice under NaCl stress (Roy and Wu, 2001). Later, they introduced the *SAMDC* gene of the durum wheat × barley hybrid Tritordeum into rice. A Southern blot analysis showed that the *SAMDC* gene was stably integrated. Under NaCl stress, the growth potential of transgenic rice seedlings was better than that of non-transgenic rice, and the contents of Spd and Spm were 3–4 times higher in transgenic lines than in non-transgenic lines (Roy and Wu, 2002). Similarly, micropropagated transgenic *Lotus tenuis* plants expressing *ADC* were healthier than wild-type plants under salinity stress and showed better osmotic adjustment (5.8-fold) (Espasandin et al., 2018). An ADC2 deletion mutant of Arabidopsis showed extreme sensitivity to salt stress, which was alleviated by applying exogenous Put (Naka et al., 2010).

### PAs and Oxidative Stress

Polyamines play a complex role in plant oxidative stress (Minocha et al., 2014). On one hand, polyamines can increase the activity of various antioxidant enzymes in plants, so that it can effectively regulate oxidative stress in plants caused by various environmental factors. Maize leaves pretreated with Spm and Put showed increased tolerance to oxidative stress induced by paraquat (Durmu and Kadioglu, 2005). Exogenous Spd significantly increased the contents of Spd and Spm and reduced the content of Put in the roots of cucumber seedlings under hypoxia stress. These changes were related to increased antioxidant enzyme activity, enhanced ROS scavenging ability, and less membrane lipid peroxidation, which ultimately led to enhanced hypoxia stress tolerance (Jia et al., 2008; Wu et al., 2018). Under cadmium- and copper-induced oxidative stress, lipid peroxidation in sunflower leaf discs increased, while the activities of glutathione reductase (GR) and superoxide dismutase (SOD) decreased (Groppa et al., 2001). When plants were treated with exogenous PAs (1 mM), Spm treatment reduced the effects of Cd^2+^ and Cu^2+^ on lipid peroxidation almost to control values (Tajti et al., 2018). In addition, GR activity was completely restored by Spm or Spd treatments, and SOD activity under Cu^2+^ treatment was restored by Spm treatment (Table 3).

On the other hand, PAs are a source of reactive oxygen species. Because their catabolism produces the strong oxidizers H_2_O_2_ and acrolein, PAs can potentially be the cause of cellular harm under stress conditions (Minocha et al., 2014). However, H_2_O_2_ is also a signaling molecule that can enter the stress signal transduction chain and activate an antioxidant defense response (Groppa and Benavides, 2008). Thus, it seems that PAs are regulators of redox homeostasis that play a dual role in plant oxidative stress (Saha et al., 2015).

### Other

Plants can also be affected by other stresses, such as acid stress, radiation stress, wounding, heavy metals (Tajti et al., 2018), and diseases and pests (Khajuria and Ohri, 2018). Few studies have focused on these topics, but current data indicate that PAs are important in the responses to these stresses. Exogenous Put was shown to regulate the balance of active oxygen metabolism under acid stress and stabilize membrane system structure, thereby protecting plants from acid stress and improving their acid resistance (Li et al., 1995). *Arabidopsis* plants subjected to mechanical injury showed increased expression of *ADC2* (Perezamador et al., 2002). Similarly, mechanical wounding of the first leaves of oilseed rape led to a significant increase in free Put content in the wounded first leaf and the unwounded second leaf (Cowley and Walters, 2010). Treatment with the heavy metals Hg^2+^ and Cr^6+^ led to reduced contents of Spd and Spm and decreased activities of SOD, catalase, and peroxidase in amaranth leaves, leading to excessive accumulation of membrane lipid peroxides (malondialdehyde) and a significant decrease in chlorophyll and soluble protein contents. Exogenous Spd ameliorated these negative effects of Hg^2+^ and Cr^6+^ (Wang et al., 2003a; Wang and Shi, 2004).

As well as being involved in abiotic stress responses, PAs are also closely related to biotic stress responses in plants. Plant tissues infected with pathogens accumulate large amounts of PAs, which inhibit the growth of bacteria and viruses and inactivate viruses. When pathogens invade plant cells, they induce PA accumulation and PA oxidase activity; this leads to increased H_2_O_2_ content, which prevents pathogens from infecting cells (Yordanova et al., 2003). Overexpression of the *ADC* gene from trifoliate orange significantly increased resistance to ulcer disease in citrus (Wang, 2009). Similarly, a higher Put content was found to be associated with greater insect resistance in Chinese cabbage (Wang, 2007).

## Conclusions and Future Prospects

This paper represents a comprehensive review of the published literature on the relationship between PAs and plant growth, development and stress tolerance. We explored the role of PAs in plant developmental processes ranging from flowering to senescence, and discussed the effects of PAs on plant growth and development. This information provides a reference for the future research on the regulation mechanism of PAs and the use of exogenous PAs to regulate plant growth in production. In recent years, many studies have focused on the relationship between PAs and plant growth and development, but most of them have been relatively simple and similar. Almost all of them have focused the effects of exogenous PAs on the growth and development of fruit or vegetable crops or model plants. However, it is becoming increasingly popular to increase endogenous PA production via genetic manipulation to regulate plant growth. There are still many questions to answer regarding the roles of PAs in regulating plant growth and development. It is still largely unknown how the biosynthetic and catabolic pathways are regulated at the transcriptional, translational, and post-transcriptional levels. Further research is required to uncover the exact mechanism of PA accumulation to improve plant stress resistance. Similarly, there is still much to learn about the metabolic relationship between PAs and other hormones during the growth and development of higher plants, especially the relationship between PAs and ethylene. With the advancement of molecular biology techniques, research is now focusing on events at the molecular level. The contents of intracellular PAs have been modulated by altering the expression of *ADC, ODC*, and *SAMDC*. The use of transgenic methods to manipulate PA metabolism has become an effective tool to study the physiological functions of PAs in higher plants. Illuminating the regulation mechanism of PAs at the molecular level should be a major research direction in the future.

## Author Contributions

DC read a lot of literatures and wrote the paper. QS provided the writing direction and revised the paper. LY provided some suggestions for the paper. AY and BZ helped in polish the language of this article.

### Conflict of Interest Statement

The authors declare that the research was conducted in the absence of any commercial or financial relationships that could be construed as a potential conflict of interest.

## Abbreviations

PAs: polyamines
F-PAs: free polyamines
CC-PAs: covalently conjugated polyamines
NCC-PAs: non-covalently conjugated polyamines
PSCC-PAs: perchloric acid-soluble covalently conjugated polyamines
PISCC-PAs: perchloric acid-insoluble covalently conjugated polyamines
Put: putrescine
Spd: spermidine
Spm: spermine
Tspm: thermospermine
Cad: cadaverine
Arg: arginine
ADC: arginine decarboxylase
Agm: agmatine
NCPA: N-carbamoylputrescine
NCPAH: N-carbamoylputreseine amidohydrolase
Orn: ornithine
ODC: ornithine decarboxylase
Cit: citrulline
CDC: citrulline decarboxylase
DFMA: difluoromethylarginine
DFMO: difluoromethylornithine
AIH: agmatine iminohydrolase
SPDS: spermidine synthase
CHA: clohexylamine, the inhibitor of SPDS
SPMS: spermine synthase
ACL5: thermospermine synthase
SAM: s-adenosylmethionine
SAMDC: S-adenosylmethionine decarboxylase
SAMS: S-adenosylmethionine synthase
ACC: 1-amino-1-carboxycyclopropane
ACS: 1-aminocyclopropane-1-carboxylate synthase
ACO: 1-aminocyclopropane-1-carboxylate oxidase
MGBG: methylglyoxal bis (guanylhy-drazone), the inhibitor of SAMDC
DeSAM: decarboxylated SAM
MTA: 5′-methyl-thioadenosine
DAO: diamine oxidase
PAO: polyamine oxidase
PYRR: pyrroline
GABA: γ-aminobutyric acid
PYRR-DH: pyrroline dehydrogenase
FAD: flavin adenine dinucleotide
Dap, 1: 3-diaminopropane
PBCP: polyamines back-conversion pathway
ABA: abscisic acid
IAA: auxin
ETH: ethylene
GA: gibberellic acid
Cyt: cytokinins
ESM: embryogenic suspensor mass
ROS: reactive oxygen species
PEG: polyethylene glycol
CAT: catalase
SOD: superoxide dismutase
GR: glutathione reductase.
